# Effects of Temperature, Light and Salt on the Production of Fucoxanthin from *Conticribra weissflogii*

**DOI:** 10.3390/md21090495

**Published:** 2023-09-16

**Authors:** Feng Li, Xiangyu Rui, David Kwame Amenorfenyo, Yao Pan, Xianghu Huang, Changling Li

**Affiliations:** College of Fisheries, Guangdong Ocean University, Zhangjiang 524088, China; 2112001098@stu.gdou.edu.cn (X.R.); davidamenorfenyo@yahoo.com (D.K.A.); 2112001076@stu.gdou.edu.cn (Y.P.); huangxh@gdou.edu.cn (X.H.)

**Keywords:** algal cultivation, *C. weissflogii*, fucoxanthin, temperature, light intensity, salinity

## Abstract

Fucoxanthin is a natural active substance derived from diatoms that is beneficial to the growth and immunity of humans and aquatic animals. Temperature, light and salinity are important environmental factors affecting the accumulation of diatom actives; however, their effects on the production of fucoxanthin in *C. weissflogii* are unclear. In this study, single-factor experiments are designed and followed by an orthogonal experiment to determine the optimal combination of fucoxanthin production conditions in *C. weissflogii.* The results showed that the optimum conditions for fucoxanthin production were a temperature of 30 °C, a light intensity of 30 umol m^−2^ s^−1^ and a salinity of 25. Under these conditions, the cell density, biomass, carotenoid content and fucoxanthin content of *C. weissflogii* reached 1.97 × 10^6^ cell mL^−1^, 0.76 g L^−1^, 2.209 mg L^−1^ and 1.372 mg g^−1^, respectively, which were increased to 1.53, 1.71, 2.50 and 1.48 times higher than their initial content. The work sought to give useful information that will lead to an improved understanding of the effective method of cultivation of *C. weissflogii* for natural fucoxanthin production.

## 1. Introduction

The new health paradigm towards disease prevention has led to an increasing focus on nutraceuticals with both nutritional and pharmaceutical benefits. Nutraceuticals made from natural derivatives are becoming more and more popular due to their safety. Natural carotenoids, naturally synthesized by algae, plants, yeast and bacteria, are rapidly emerging in nutraceuticals and functional foods [1]. Fucoxanthin is a highly valuable oxygenated carotene [2] that accounts for about 10% of the total carotenoid production on Earth, and is considered to be the most abundant but almost untapped carotenoid resource [3]. Fucoxanthin has many beneficial effects on human and animal health, including antioxidant, anti-inflammatory, anti-obesity, anti-diabetic effects, etc. [2,4], with great scientific and economic potential in the fields of nutraceuticals, biopharmaceuticals and functional foods. It is estimated that the global fucoxanthin market is expected to reach more than USD 100 million over the next 2 years [5].

Presently, the main source of commercialization of fucoxanthin is brown seaweeds [6]. However, brown seaweeds cannot meet the needs of the global market for fucoxanthin due to their slow growth, low yield, high cost and quality problems related to heavy metal pollution [7]. Compared with brown seaweeds, photosynthetic single-cell microalgae are considered to be an ideal alternative and a sustainable source of fucoxanthin in commercial production due to their rapid growth, richer fucoxanthin content, shorter doubling time and easy cultivation in photobioreactors [2,3,7]. However, there are still many bottlenecks in producer screening, optimization of cultivation conditions and biosynthetic pathway steps, resulting in the commercial production of microalgal fucoxanthin still being in its infancy [3].

Marine diatoms are rich in fucoxanthin, which can contain up to 100 times more than brown seaweeds [7], and are considered to be more promising organisms for the production of fucoxanthin [6]. It is estimated that there are about 200,000 species of diatoms in nature [1]. However, in the last decade, only about 40 species of diatoms used to synthesize fucoxanthin have been developed and characterized [3]. *Conticribra weissflogii* is a typical marine planktonic diatom which has the characteristics of fast growth, strong adaptability, being rich in polyunsaturated fatty acids, etc., and is often used as live feed by aquaculturists for large-scale cultivation [8,9]. *C. weissflogii* is competitive as a potential fucoxanthin producer, driven by the success of its large-scale cultivation. However, the optimization of abiotic parameters for fucoxanthin production from *C. weissflogii* remain a big challenge. In this work, the effects of temperatures, light intensities and salinities on the cell density, biomass, carotenoid content and fucoxanthin content of *C. weissflogii* were studied, and an attempt was made to determine the optimal combination for the abiotic parameters of fucoxanthin production via orthogonal experiments. These results are valuable for optimizing the abiotic parameters of fucoxanthin production by *C. weissflogii*.

## 2. Results

### 2.1. Effects of Temperature on Cell Density, Biomass, Carotenoid Content and Fucoxanthin Content of C. weissflogii

**Figure 1:**
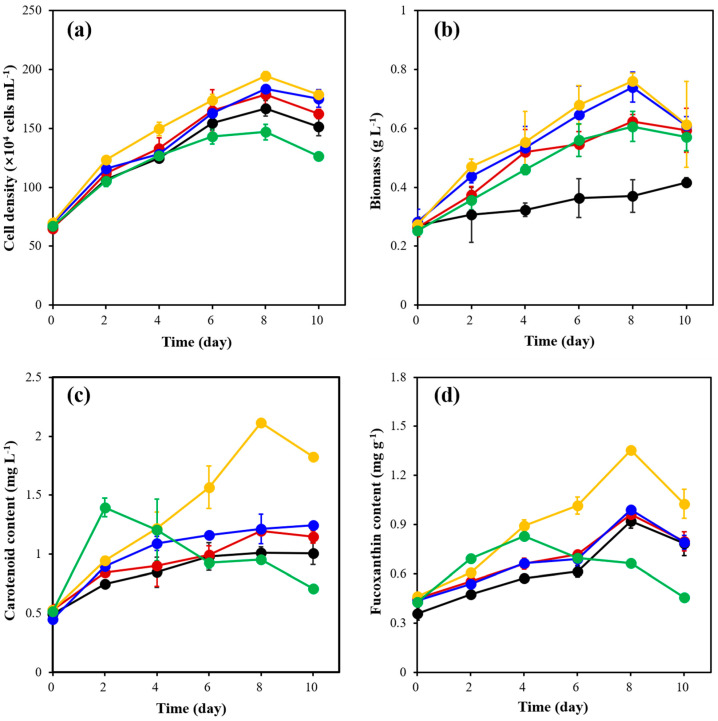
The changes in cell density (**a**), biomass (**b**), carotenoid content (**c**) and fucoxanthin content (**d**) of *C. weissflogii* cultures in 15 °C (black), 20 °C (red), 25 °C (blue), 30 °C (yellow) and 35 °C (green) groups.

The results showed that the maximum cell density of *C. weissflogii* (Figure 1a) in each experimental group was reached on the 8th day of cultivation. However, the cell density in the 30 °C group was significantly higher than the other experimental groups, with a maximum cell density of 1.94 ± 0.04 × 10^6^ cell mL^−1^, which was 1.81 times higher than the initial cell density. Also, it can be observed from Figure 1b that the biomass of all experimental groups, except the 15 °C group, reached their maximum value on the 8th day. On day 8, the biomass of the 30 °C group was 0.76 ± 0.03 g L^−1^, which was significantly higher among the groups.

From Figure 1c, the carotenoid content of all experimental groups, except for that of the 30 °C and 35 °C groups, experienced a continuous growth rate. However, the carotenoid content in the 30 °C group was significantly higher compared with the other experimental groups with a maximum value of 2.11 ± 0.02 mg L^−1^, which was 2.99 times higher than the initial content (0.53 ± 0.02 mg L^−1^). Similarly, the fucoxanthin content in the 30 °C group was significantly higher, with a maximum of 1.36 ± 0.02 mg g^−1^. The results showed that different temperatures had significant effects on the cell density, biomass, carotenoid content and fucoxanthin content of *C. weissflogii*.

**Figure 2:**
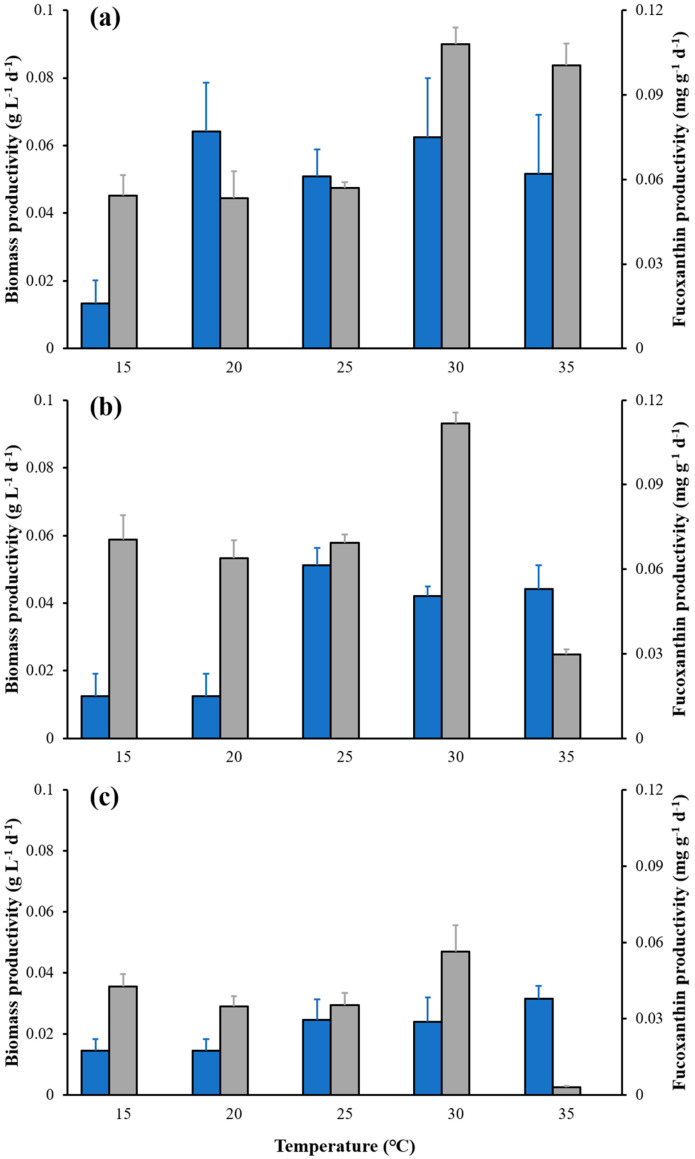
Effect of temperature on biomass productivity (blue bars) and fucoxanthin productivity (gray bars) at days 4 (**a**), 8 (**b**) and 10 (**c**) of cultures at the different temperature, respectively. The data are mean ± standard deviation.

The maximum biomass productivity of the 15 °C group was less than 0.015 g L^−1^ d^−1^ for the 10-day cultivation periods (Figure 2). The biomass productivity in groups 20-, 30- and 35 °C reached a maximum after 4 days of cultivation (Figure 2a), and then the value declined. The maximum biomass productivity in the 25 °C group occurred on the 8th day of cultivation (Figure 2b). The maximum fucoxanthin productivity in the 35 °C group was reached after 4 days of cultivation. However, the maximum fucoxanthin productivity in 15-, 20-, 25- and 30 °C groups all occurred on day 8 (Figure 2b). A maximum value of 0.11 mg g^−1^ d^−1^ of fucoxanthin productivity was obtained at 30 °C, which was 1.12–1.75 times higher than the other experimental groups.

### 2.2. Effects of Light Intensity on Cell Density, Biomass, Carotenoid Content and Fucoxanthin Content of C. weissflogii

**Figure 3:**
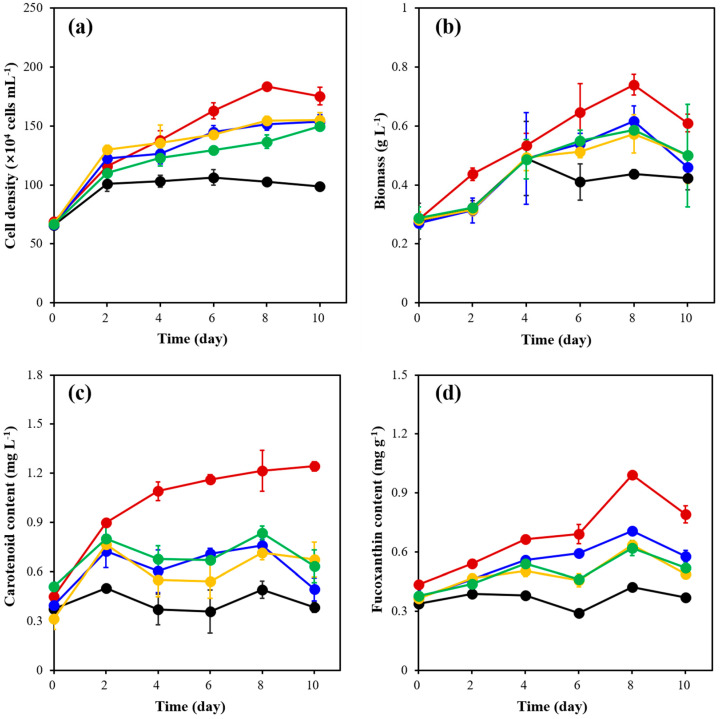
The cell density (**a**), biomass (**b**), carotenoid content (**c**) and fucoxanthin content (**d**) of *C. weissflogii* cultures in 0 μmol m^−2^ s^−1^ (black), 30 μmol m^−2^ s^−1^ (red), 60 μmol m^−2^ s^−1^ (blue), 90 μmol m^−2^ s^−1^ (yellow) and 120 μmol m^−2^ s^−1^ (green) groups.

The results (Figure 3a) showed that, except for the experimental groups of 0 μmol m^−2^ s^−1^ and 30 μmol m^−2^ s^−1^, the cell density of the other three groups of *C. weissflogii* experienced continuous growth. However, the maximum cell density was observed on the 10th day. The *C. weissflogii* showed maximum algal cell density on the 8th day under 30 μmol m^−2^ s^−1^, with 1.84 ± 0.03 × 10^6^ cell mL^−1^, which was 1.67 times higher than the initial algal cell density. As shown in Figure 3b, the maximum biomass of all other experimental groups except for the 0 μmol m^−2^ s^−1^ appeared on the 8th day. However, the biomass in the 30 μmol m^−2^ s^−1^ group was the maximum in all groups with 0.74 ± 0.03 g L^−1^, which was 1.64 times higher than the initial content (0.28 ± 0.03 g L^−1^).

It can be seen from Figure 3c that the content of carotenoids in each experimental group showed different peaks on different days, except for the 30 μmol m^−2^ s^−1^ group, which showed continuous growth. The maximum value appeared on the 10th day of 30 μmol m^−2^ s^−1^. At this time, the content of carotenoids in *C. weissflogii* was 1.24 ± 0.03 mg L^−1^, which was 1.76 times higher than the initial content (0.45 mg L^−1^). According to Figure 3d, except for the 0 μmol m^−2^ s^−1^ group, the fucoxanthin content of the other experimental groups increased first and then decreased, and the maximum value appeared on the 8th day. At this time, the fucoxanthin content of the 30 μmol m^−2^ s^−1^ experimental group was 0.99 ± 0.01 mg g^−1^, which was 1.27 times higher than the initial content (0.44 ± 0.01 mg g ^−1^). It was proved that different light intensities had significant effects on the cell density, biomass, carotenoid content and fucoxanthin content of *C. weissflogii*.

**Figure 4:**
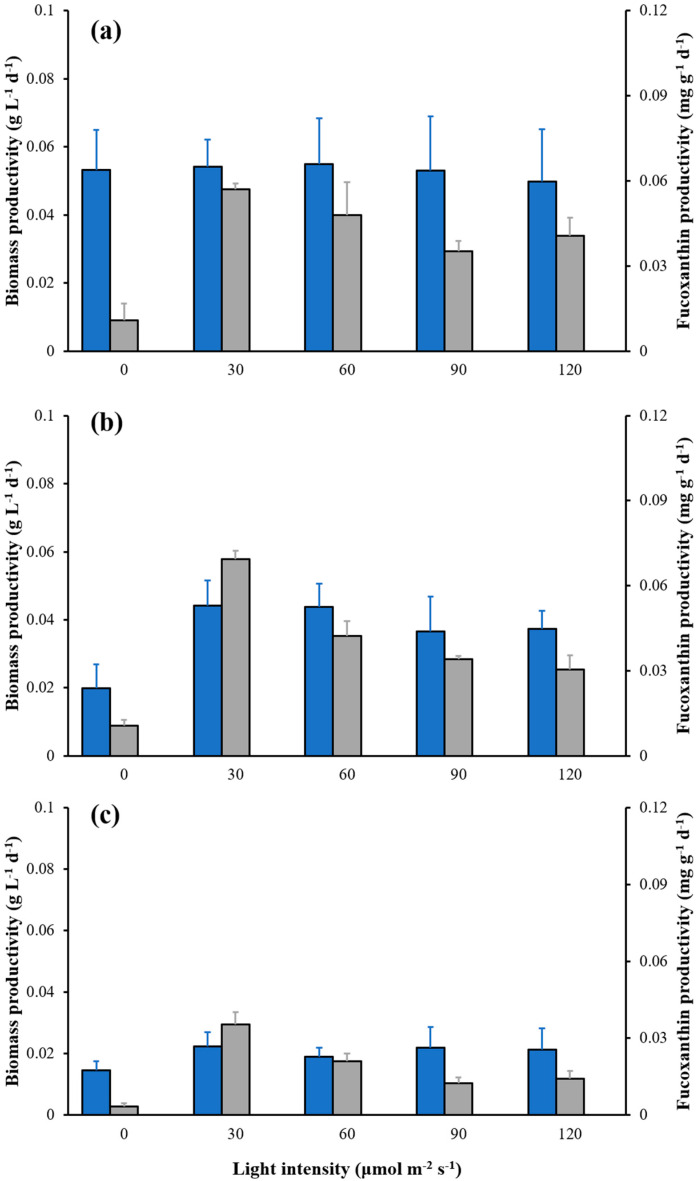
Effect of light intensity on biomass productivity (blue bars) and fucoxanthin productivity (gray bars) at days 4 (**a**), 8 (**b**) and 10 (**c**) of cultures with different light intensities, respectively. The data are mean ± standard deviation.

The biomass productivity in all light intensity groups reached a maximum after 4 days of cultivation (Figure 4a); the values declined steadily, and finally reached the lowest value on the 10th day of cultivation (Figure 4c). The *C. weissflogii* experienced maximum fucoxanthin productivity under 0, 60, 90 and 120 μmol m^−2^ s^−1^ after day 4 of the cultivation period, while under 30 μmol m^−2^ s^−1^, the maximum productivity of fucoxanthin was obtained until the 8th day of cultivation (Figure 4b). However, the fucoxanthin productivity value declined sharply after the 8th day of cultivation. From the results, 30 μmol m^−2^ s^−1^ showed the maximum value of fucoxanthin productivity compared with other treatments (0, 60, 90 and 120 μmol m^−2^ s^−1^) with a maximum value of 0.07 mg g^−1^ d^−1^. Therefore, 30 μmol m^−2^ s^−1^ could serve as the optimum light intensity for the maximum production of fucoxanthin. (Figure 4b,c).

### 2.3. Effects of Salinity on Cell Density, Biomass, Carotenoid Content and Fucoxanthin Content of C. weissflogii

**Figure 5:**
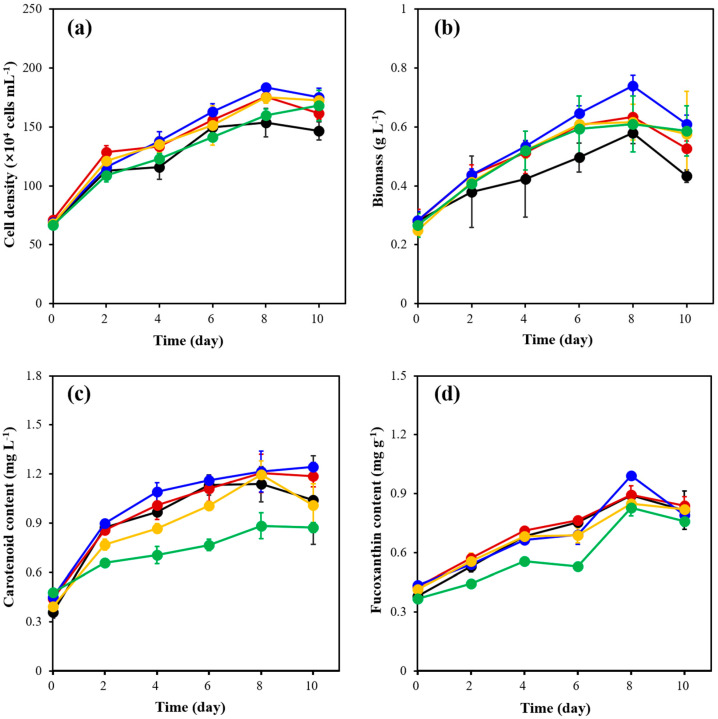
The cell density (**a**), biomass (**b**), carotenoid content (**c**) and fucoxanthin content (**d**) of *C. weissflogii* cultures in 15 ‰ (black), 20 ‰ (red), 25 ‰ (blue), 30 ‰ (yellow) and 35 ‰ (green) groups.

As shown in Figure 5a, the cell density of *C. weissflogii* in all experimental groups except the 35‰ group increased on the 2nd day but decreased on the 4th day. On the 8th day of cultivation, the 25‰ group obtained the highest cell density (1.84 ± 0.03 × 10^6^ cell mL^−1^) compared with other experimental groups. A similar trend was observed in biomass production (Figure 5b). The maximum biomass (0.74 ± 0.03 g L^−1^) was obtained in the 25‰ group, which was significantly (*p* < 0.05) higher than the other experimental groups.

It can be observed from Figure 5c that the 25‰ group showed a higher carotenoid content of *C. weissflogii* compared with the other experimental groups with 1.24 ± 0.03 mg L^−1^, which was 1.76 times higher than the initial value of 0.45 ± 0.04 mg L^−1^. According to Figure 5d, the highest fucoxanthin content of *C. weissflogii* was observed in the 25‰ group. The maximum value of fucoxanthin content was reached, 0.99 ± 0.01 mg g^−1^, on the 8th day of cultivation, which was 1.25 times higher than the initial content (0.44 ± 0.01 mg g^−1^). The results demonstrated that different salinities had significant effects on the cell density, biomass, carotenoid content and fucoxanthin content of *C. weissflogii*.

**Figure 6:**
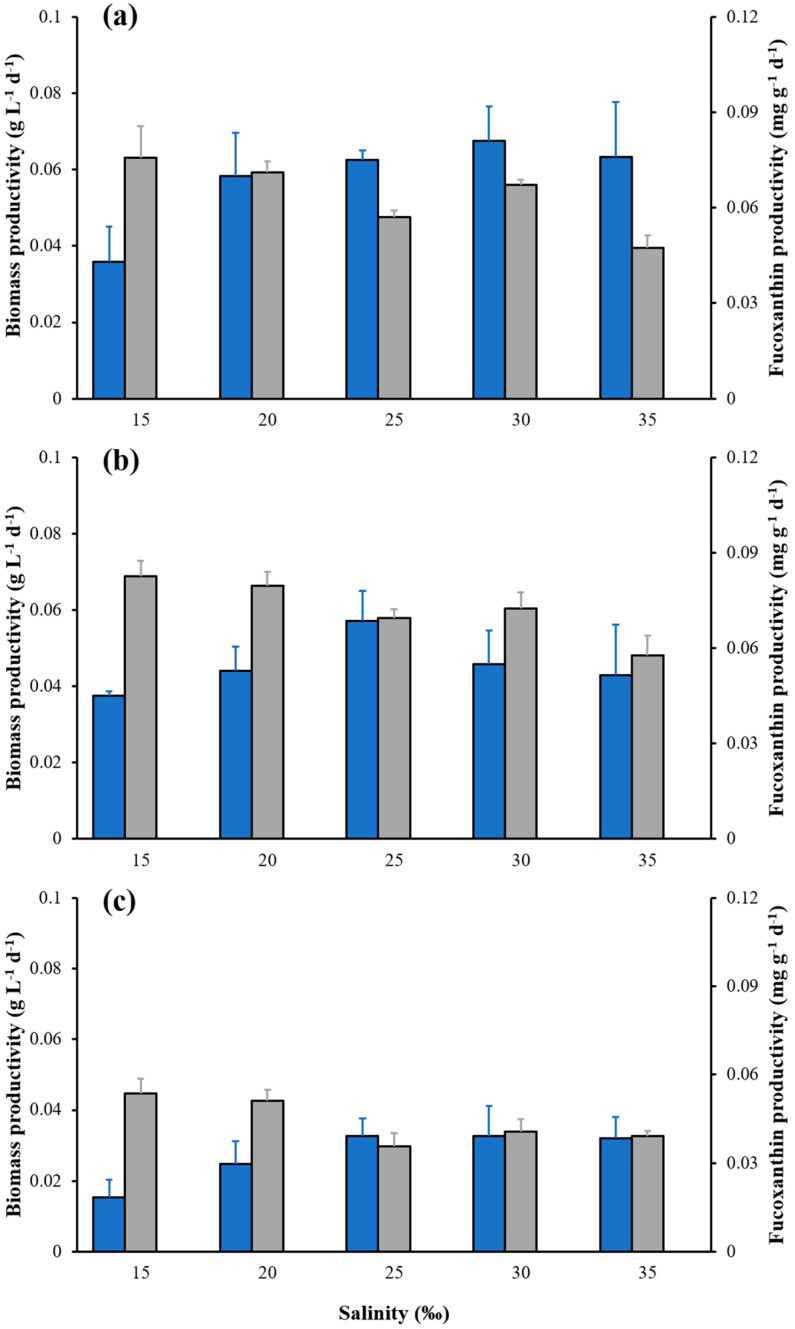
Effect of salinity on biomass productivity (blue bars) and fucoxanthin productivity (gray bars) at days 4 (**a**), 8 (**b**) and 10 (**c**) in cultures with different salinities, respectively. The data are mean ± standard deviation.

From Figure 6a, all salinity groups except the 15 ‰ group showed a sharp decline in biomass productivity after the 8th day of cultivation. The fucoxanthin productivity under salinity 15 ‰ was significantly higher than other salinity groups, with a maximum value of 0.83 mg g^−1^ d^−1^ (Figure 6b).

### 2.4. The Effect of Orthogonal Design Test on Cell Density, Biomass, Carotenoid Content and Fucoxanthin Content of C. weissflogii

The effects of temperature, light intensity and salinity on the cell density, biomass, carotenoid content and fucoxanthin content of *C. weissflogii* were preliminarily determined using single-factor experiment. In order to obtain the best conditions of fucoxanthin production from *C. weissflogii*, three optimal gradients were selected from the five gradients of temperature, light and salinity to construct an L9 (3^3^) orthogonal test (Table 1). According to the results in Table 2, the optimal combination conditions were: temperature 30 °C, light intensity 30 μmol m^−2^ s^−1^, 25‰. In this combination, the maximum values of all four parameters were significantly higher than those of the other groups, with a maximum cell density of 1.97 × 10^6^ cell mL^−1^, a maximum biomass of 0.76 g L^−1^, a maximum carotenoid content of 2.209 mg L^−1^ and a maximum fucoxanthin content of 1.372 mg g^−1^.

The range analysis of biomass is shown in Table 3. The range values (R) of temperature, light intensity and salinity on biomass were 0.14, 0.06 and 0.06, respectively, so the most important factors affecting the growth of cell dry weight are temperature, followed by light intensity and salinity. From Table 4, the range values of temperature, light intensity and salinity on fucoxanthin content were 0.606, 0.098 and 0.280, respectively. It is therefore clear that the most important factors affecting the growth of fucoxanthin content are temperature, followed by salinity and, finally, light intensity.

## 3. Discussion

Temperature is one of the most important environmental factors affecting the growth rate, biochemical composition and nutrient requirements of microalgae [10]. The tolerance of different microalgae species to temperature is also quite different [11]. Due to the different growth requirements of various algae, they all have their own temperature adaptation ranges [12]. For example, *Chaetoceros muelleri* can grow and reproduce normally in the range of 25–34 °C. The suitable growth temperature range is 28–31 °C, and the optimum growth temperature is 31 °C [13]. The growth rate of *Skeletonema munzelii* was significantly different at different temperatures. It could grow normally at 10–25 °C, and the optimum temperature was 25 °C [14]. The suitable temperature range of *Nitzschia closterium* is 10–25 °C [15]. The suitable growth temperature of *Chlorella* is 20–35 °C. Temperatures below or above the optimum range could affect the growth and metabolic actives of microalgae [16]. Li et al. [17] reported a suitable temperature range of 26–32 °C for large-scale cultivation of *C. weissflogii* in the summer. Similar conclusions were observed in our study, that a temperature condition of 30 °C is most favorable for the growth and fucoxanthin accumulation of *C. weissflogii*. After 8 days of cultivation at this temperature, the cell density, biomass, carotenoid content and fucoxanthin content of *C. weissflogii* were higher than those of the other temperature experimental groups.

Marine microalgae are mainly autotrophic through light energy, and the efficiency of photosynthesis plays a vital role in the growth of algae. Therefore, light intensity could directly affect the growth rate, photosynthetic activities and biochemical components of microalgae [18,19,20]. Studies have shown that in a certain light range, with the increase in illumination, the photosynthetic efficiency of microalgae also increases correspondingly, but when the light intensity exceeds its saturation point, photosynthesis will weaken or even inhibit the growth of microalgae [21]. Optimum light intensity is highly dependent on the microalgal species. For example, the optimum light intensity of *Skeletonema costatum* could be higher than 120 μmol m^−2^ s^−1^ [22]. The optimum light intensity of *Isochrysis galbana* was reported to be 150–200 μmol m^−2^ s^−1^ [23]. The algal cell density of *C. muelleri* was significantly higher than other treatments under optimum light (200 μmol m^−2^ s^−1^) intensity [13]. However, in some astaxanthin-producing microalgae, the light intensity conditions required for growth and astaxanthin accumulation are different. For example, *Haematococcus pluvialis* is suitable for growth in low-light conditions (<100 μmol m^−2^ s^−1^), while astaxanthin requires strong light conditions (>200 μmol m^−2^ s^−1^) to achieve more efficient accumulation [24]. Since fucoxanthin is a light-harvesting pigment, a higher light intensity leads to a decrease in fucoxanthin content [10]. Previous studies have shown that the optimal light intensity range for inducing fucoxanthin accumulation is 10–100 μmol m^−2^ s^−1^ [10], and the highest content of fucoxanthin can reach 4.28% of dry weight under low light induction in this range [25]. In this study, *C. weissflogii* was cultured in a light intensity of 0–120 μmol m^−2^ s^−1^. The results showed that when the light intensity was 30 μmol m^−2^ s^−1^, the algal cell density, biomass, carotenoid content and fucoxanthin content reached the maximum. This also confirmed the findings of Li et al. [17]. *C. weissflogii* is a kind of microalgae that is easy to culture under low-light conditions.

Salinity can affect the osmotic pressure, nutrient absorption and suspension of algae to a certain extent. When the salinity of the aquaculture water where the microalgae cells are located changes, the osmotic pressure of the cells will also change [26]. The microalgae cells experience different degrees of damage when the low salinity is above or below the optimum range [27]. Like temperature and light intensity, optimum salinity is also highly dependent on the microalgal species [10]. In some fucoxanthin-producing microalgae, they prefer a specific salinity to strive for growth and produce a higher amount of fucoxanthin. For example, *Isochrysis galbana* exhibited a higher growth and fucoxanthin content than 20‰ salinity at 35‰ salinity [28]. An optimization study showed that after culturing microalgae at four different salinities (5, 10, 20 and 30‰), the growth of *Phaeodactylum tricornutum* and fucoxanthin content was optimal at salinity levels of 20‰ [29]. The other microalgae, *Cylindrotheca fusiformis*, was observed to grow best at a salinity of 30‰, while fucoxanthin content was the highest at a salinity of 10‰ [29]. In this study, the 25‰ group showed higher biomass and fucoxanthin content after 8 days of cultivation compared with the other four salinity experimental groups. This result was consistent with the results of Chen et al. [30]. In addition, it was also observed in this work that carotenoid and fucoxanthin accumulation was inhibited when salinity reached 35‰. These results highlighted that optimizing microalgae salinity levels can promote microalgae biomass concentration and fucoxanthin content.

## 4. Materials and Methods

### 4.1. C. weissflogi Strain and Growth Conditions

The microalgae *C. weissflogi* was isolated and purified from a shrimp pond in southern China in 2019 by the Laboratory of Algal Resources Development and Ecological Remediation of Aquaculture Environment, Guangdong Ocean University, Zhanjiang, China. Stock cultures of *C. weissflogi* were maintained at 25 ± 1 °C under a continuous light intensity of 30 μmol m^−2^ s^−1^ in 1 L flasks. A modified version of F/2 media was used, and the detailed formulation can be found in our recent study [31]. We centrifuged and collected 10-day-old algal cells for experiments.

### 4.2. Single-Factor Experiment

Five concentration gradients were set up for each experiment group in single-factor experiments of temperature (15, 20, 25, 30 and 35 °C), light intensity (0, 30, 60, 90 and 120 μmol m^−2^ s^−1^) and salinity (15, 20, 25, 30 and 35‰). Temperature single-factor experiments were performed at 30 μmol m^−2^ s^−1^ and 25‰. Light single-factor experiments were performed at 25 °C and 25‰. Salinity single-factor experiments were performed at 25 °C and 30 μmol m^−2^ s^−1^. The culture volume for all cultures was 700 mL, and the initial algae density was about 6 × 10^5^ cell mL^−1^. During the experiment, all cultures were continuously illuminated, sealed and not inflated. Three parallels were set for each treatment, and the experiment period was 10 days.

### 4.3. Orthogonal Experimental Design

According to the results of single-factor experiments, three optimal gradients were selected from the five gradients of temperature, light and salinity, and a three-factor three-level orthogonal experiment was constructed with the aim of determining the optimal combination of temperature, light and salinity. The orthogonal experiment consists of a total of nine groups, each with three parallels. The specific operation steps are the same as above.

### 4.4. Cell Density

The cell density of *C. weissflogii* was counted using a Neubauer improved cell counting chamber (25 mm × 16 mm) under a BX53 fluorescence microscope after being fixed with Lugol’s iodine solution [32].

### 4.5. Biomass

Biomass was determined using the drying differential weight method [33]. Filter 10 mL of algae suspension through a pre-weighed acetate membrane (M_1_). Wash twice with distilled water in this process. The membrane containing algal cells is then placed in an oven and dried at 90 °C for 500 min to constant weight. The total mass M_2_ is measured and recorded. The biomass content was calculated according to the following formula.

DW (Dry weight) = (M_2_−M_1_) × 10^3^/10 (1)

### 4.6. Pigment Content

Using ethanol extraction method [34], take 10 mL of algal liquid, centrifuge at 5000 r min^−1^ for 10 min, discard the supernatant, add 10 mL of ethanol with a volume fraction of 95%, dark-treat 24 h, centrifuge at 5000 r min^−1^ for 10 min and measure the supernatant with a spectrophotometer The optical density value D of the solution was measured at 480, 510 and 750 nm. The content of carotenoids was calculated according to the following formula.

ρ_(Carotenoids)_ = 7.6 × [(D_480_−D_750_) − 1.49 × (D_510_−D_750_)] (2)

Extraction with organic solvents [35]. Take 80 mL of the culture, freeze-dry it in a freeze-dryer for 24 h after centrifugation (5000 r min^−1^, 10 min) and remove the supernatant. Add absolute ethanol to make a material-to-liquid ratio of 1 g:40 mL, and then extract twice for 1 h each while protected from light at 60 °C. After leaching, the supernatant is collected via centrifugation (5000 r min^−1^, 10 min) and the absorbance (D_445_) is determined with a UV spectrophotometer at 445 nm. The fucoxanthin content C can be expressed as:

C_(Fucoxanthin)_ = (1000 × D_445_ × N × V)/(A′ × M × 100) (3)

In the formula, N is the dilution ratio; V is the volume of the crude extract; A′ is the theoretical absorption value of a solute with a volume fraction of 1% in a colorimetric cup of 1 cm optical range length and has a value of 1600; and M is the sample mass.

### 4.7. Data Analysis

Excel 2019 was used to process the data and draw charts. SPSS 26 was used to analyze the single-factor analysis of variance and orthogonal range analysis of each index to test the differences between the data of each group. *p* < 0.05 indicated that there was a significant difference.

## 5. Conclusions

In this study, the single-factor experiment and orthogonal experiment were used to optimize the biomass and fucoxanthin production conditions of *C. weissflogii*. Settings of 30 °C, 30 μmol m^−2^ s^−1^ and 25‰ were determined to be the optimum condition for biomass and fucoxanthin production. After 8 days of cultivation under these conditions, the cell density, biomass, carotenoid content and fucoxanthin content of *C. weissflogii* reached 1.97 × 10^6^ cell mL^−1^, 0.76 g L^−1^, 2.209 mg L^−1^ and 1.372 mg g^−1^, respectively, which were increased to 1.53, 1.71, 2.50 and 1.48 times higher than their initial content. Cultivated environmental factor optimization improved biomass and fucoxanthin production, but the efficient production of fucoxanthin by *C. weissflogii* still required a lot of work to do..

## Figures and Tables

**Table 1:** Orthogonal experimental design.

Group	Temperature (°C)	Light Intensity (μmol m^−2^ s^−1^)	Salinity (‰)
1	20	30	15
2	20	60	25
3	20	90	20
4	25	30	20
5	25	60	15
6	25	90	25
7	30	30	25
8	30	60	20
9	30	90	15

**Table 2:** The cell density, biomass, carotenoid content and fucoxanthin content of *C. weissflogii* on day 8 of cultivation in the orthogonal experiment.

Group	Cell Density (×10^4^ Cell mL^−1^)	Biomass (g L^−1^)	Carotenoid (mg L^−1^)	Fucoxanthin (mg g^−1^)
1	143 ± 6.42	0.53 ± 0.02	0.814 ± 0.03	0.629 ± 0.04
2	153 ± 2.89	0.52 ± 0.02	0.956 ± 0.03	0.619 ± 0.04
3	140 ± 1.53	0.56 ± 0.03	0.766 ± 0.03	0.602 ± 0.06
4	136 ± 6.56	0.54 ± 0.03	0.929 ± 0.01	0.710 ± 0.05
5	150 ± 2.52	0.53 ± 0.04	0.708 ± 0.02	0.690 ± 0.01
6	170 ± 2.89	0.53 ± 0.05	0.732 ± 0.01	0.683 ± 0.03
7	197 ± 5.77	0.76 ± 0.07	2.209 ± 0.07	1.372 ± 0.04
8	160 ± 2.52	0.67 ± 0.07	1.430 ± 0.06	1.160 ± 0.05
9	167 ± 1.73	0.57 ± 0.04	1.398 ± 0.10	1.134 ± 0.07

**Table 3:** Range analysis of biomass (g L^−1^).

Item	Level	Temperature	Light Intensity	Salinity
K-value	1	1.61	1.83	1.63
	2	1.60	1.72	1.77
	3	2.00	1.66	1.81
K-average	1	0.54	0.61	0.54
	2	0.53	0.57	0.59
	3	0.67	0.55	0.60
R		0.14	0.06	0.06
Extreme order		1	2	3

R is the range value of the factor.

**Table 4:** Range analysis of fucoxanthin content (mg g^−1^).

Item	Level	Temperature	Light Intensity	Salinity
K-value	1	1.848	2.711	1.832
	2	2.030	2.469	2.472
	3	3.666	2.419	2.672
K-average	1	0.616	0.904	0.611
	2	0.694	0.823	0.824
	3	1.222	0.806	0.891
R		0.606	0.098	0.280
Extreme order		1	3	2

R is the range value of the factor.

## Data Availability

Data is contained within the article.
